# A Multidimensional Approach to Post-concussion Symptoms in Mild Traumatic Brain Injury

**DOI:** 10.3389/fneur.2018.01113

**Published:** 2018-12-19

**Authors:** Suzanne Polinder, Maryse C. Cnossen, Ruben G. L. Real, Amra Covic, Anastasia Gorbunova, Daphne C. Voormolen, Christina L. Master, Juanita A. Haagsma, Ramon Diaz-Arrastia, Nicole von Steinbuechel

**Affiliations:** ^1^Department of Public Health, Erasmus MC, University Medical Center Rotterdam, Rotterdam, Netherlands; ^2^Institute of Medical Psychology and Medical Sociology, Georg-August-University, Göttingen, Germany; ^3^Department of Pediatrics, University of Pennsylvania Perelman School of Medicine, Philadelphia, PA, United States; ^4^Department of Emergency Medicine, Erasmus Medical Center Rotterdam, Rotterdam, Netherlands; ^5^Department of Neurology, University of Pennsylvania Perelman School of Medicine, Philadelphia, PA, United States

**Keywords:** mild traumatic brain injury, post-concussion symptoms, outcome, diagnosis, etiology, prevalence, treatment

## Abstract

Mild traumatic brain injury (mTBI) presents a substantial burden to patients, families, and health care systems. Whereas, recovery can be expected in the majority of patients, a subset continues to report persisting somatic, cognitive, emotional, and/or behavioral problems, generally referred to as post-concussion syndrome (PCS). However, this term has been the subject of debate since the mechanisms underlying post-concussion symptoms and the role of pre- and post-injury-related factors are still poorly understood. We review current evidence and controversies concerning the use of the terms post-concussion symptoms vs. syndrome, its diagnosis, etiology, prevalence, assessment, and treatment in both adults and children. Prevalence rates of post-concussion symptoms vary between 11 and 82%, depending on diagnostic criteria, population and timing of assessment. Post-concussion symptoms are dependent on complex interactions between somatic, psychological, and social factors. Progress in understanding has been hampered by inconsistent classification and variable assessment procedures. There are substantial limitations in research to date, resulting in gaps in our understanding, leading to uncertainty regarding epidemiology, etiology, prognosis, and treatment. Future directions including the identification of potential mechanisms, new imaging techniques, comprehensive, multidisciplinary assessment and treatment options are discussed. Treatment of post-concussion symptoms is highly variable, and primarily directed at symptom relief, rather than at modifying the underlying pathology. Longitudinal studies applying standardized assessment strategies, diagnoses, and evidence-based interventions are required in adult and pediatric mTBI populations to optimize recovery and reduce the substantial socio-economic burden of post-concussion symptoms.

## Introduction

**Mild traumatic brain injuries (mTBI)** are among the most common neurologic conditions, representing a substantial burden in adults and children (1–3). A subset of mTBI patients suffers from acute **post-concussion symptoms** that may manifest as somatic symptoms (e.g., nausea, dizziness, headache, blurred vision, auditory disturbance, and fatigue), cognitive complaints (memory and executive function), emotional, and/or behavioral problems (e.g., disinhibition and emotional lability) (4–6).

In 10–25% of mTBI patients, post-concussion symptoms persist over time (7–10), which is often referred to as **post-concussion *syndrome* (PCS)**. PCS is usually diagnosed according to the International Classification of Diseases (ICD)-10 (5), or following Diagnostic and Statistical Manual of Mental Disorders (DSM)-IV criteria (6). However, over the last 15 years the concept of PCS as a reliably identifiable, unique syndrome has been questioned (11, 12). Therefore, we will use the term post-concussion symptoms to describe symptoms following mTBI and will refer to *persistent* post-concussion symptoms when these persist for at least 3 months after TBI.

**Figure 1:**
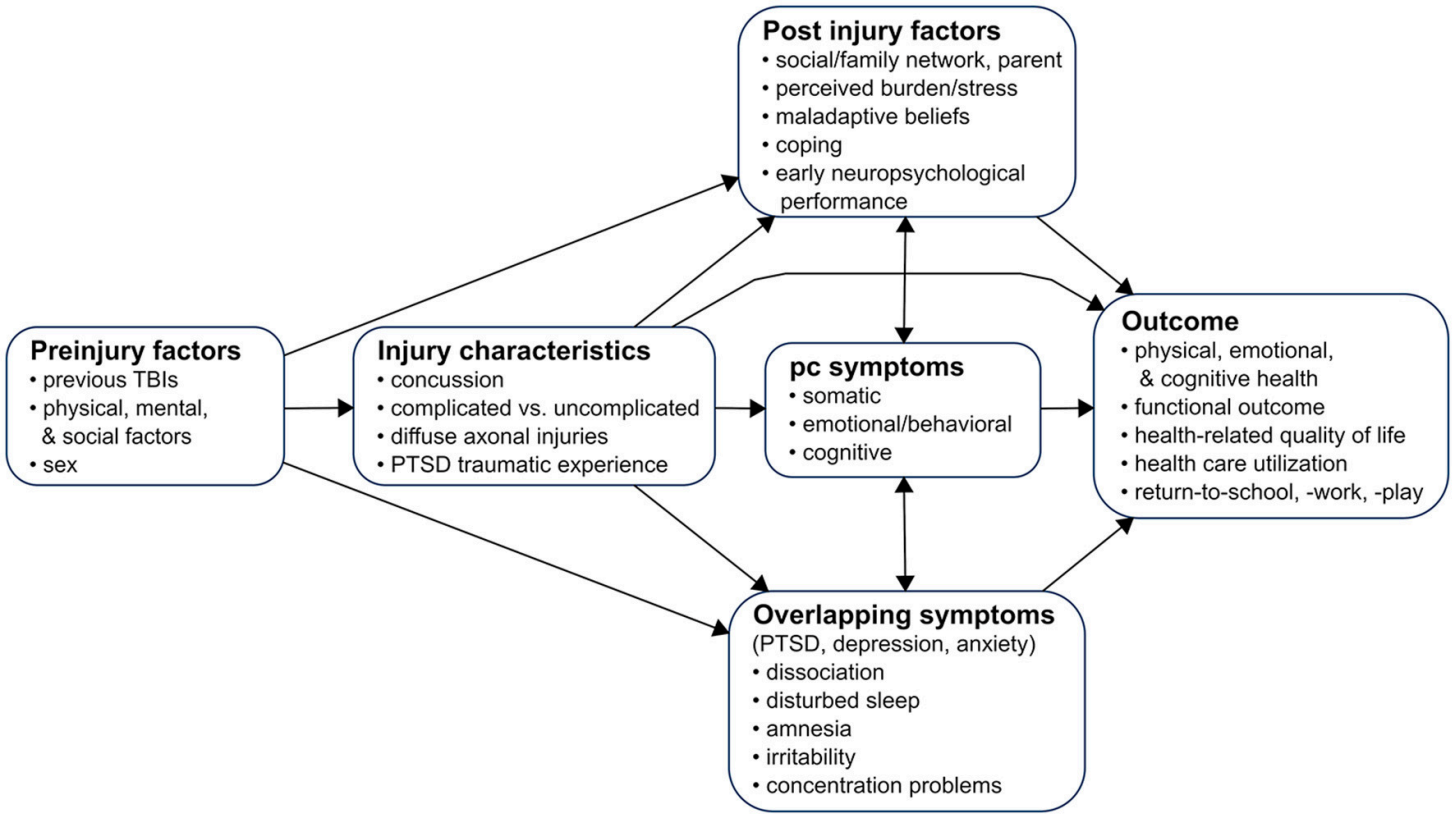
A model for the study of post-concussion symptoms after mTBI. Permission has been obtained to model our figure based on Yeates (13), © The International Neuropsychological Society 2010, published by Cambridge University Press.

This focused review (based on a systematic literature search until March 1st 2018, see Appendix A) summarizes current knowledge on epidemiology, controversies, etiology, assessment and treatment of post-concussion symptoms in adults and children. Understanding the various factors leading to post-concussion symptoms, and the complex interactions between temporal onset, biological, psychological and social factors, as well as the relative influence of injury-related and non-injury related factors, may contribute toward a better understanding, diagnosis and classification of post-concussion symptoms. Figure 1 shows current topics in research on post-concussion symptoms. In addition, an insight into the wide range of assessment methods and possible treatments may provide guidance for both clinicians (e.g., physician, psychologist, neuropsychologist, neurosurgeon, nurse, physical therapist, and occupational therapist), social worker and policy-makers.

## Materials and Methods

### Search Strategy and Selection Criteria

We searched EMBASE and MEDLINE from Jan 1th, 2010 to March 1st 2018, where landmark papers with earlier dates were also integrated. We included papers published in peer-reviewed English language journals, identifying observational, experimental, and intervention studies and reviews in civilian mTBI patients with short- and long-term (3–36 m) post-concussion symptoms or syndrome. See Appendix A for the EMBASE search strategy. Additional papers were identified by screening reference lists and citation indices and from authors' own files.After removal of duplicates, articles were excluded on the basis of title and abstract by two reviewers (MC and DV).

To warrant a minimum level of quality, papers were selected for current review if they were either (systematic) reviews or prospective cohort studies that included ≥100 patients. Exceptions were imaging studies, where lower numbers of patients were allowed and studies about treatment, where we prioritized (randomized) controlled trials. If these studies were not available, we used either retrospective or case-control studies, studies with lower number of patients or papers published before 2010. In cases where included papers did not meet our quality threshold [review, prospective cohort ≥100 patients or randomized controlled trials (RCT)], this was explicitly mentioned.

## Definitions and Epidemiology

### Mild Traumatic Brain Injury (mTBI)

The American Congress of Rehabilitation Medicine (ACRM) (4) defines mTBI as an “acute brain injury resulting from mechanical energy to the head from external physical forces,” with any of the following symptoms: loss of consciousness (LOC) not exceeding 30 min, post-traumatic amnesia (PTA) of no more than 24 h, a score of no <13 on the Glasgow Coma Scale (GCS) after 30 min post injury (or upon presentation) (14), and an (unspecified) period of confusion (feeling dazed, disoriented, and confused), or other transient neurologic abnormalities such as focal signs or seizures.

Most mTBI patients do not show trauma-related abnormalities on computed tomography (CT) scans. However, the literature on mTBI frequently distinguishes between *complicated* and *uncomplicated* mTBI and the term *complicated mTBI* is often used to refer to e.g., 5–10% of emergency department (ED) patients (15) who show abnormalities, such as subarachnoid hemorrhage, intracranial contusions, or small extra-axial hematomas. The prevalence of pediatric mTBI based on emergency department visits are likely underestimated in childhood as studies have demonstrated that most children initially seek care with their primary care doctor for these mild injuries (16). In children, findings on CT are even more rare (17) and multiple effective clinical prediction rules have been developed to reduce unnecessary CT use in children (18). Special consideration should be given for children <2 years of age with regard to decision-making about the use of CT scans in the setting of head trauma.

### Diagnosis of Post-concussion Syndrome

PCS is usually defined according to DSM-IV or ICD-10 criteria, which both focus on symptom presentation (19). These manuals agree on the prerequisite history of brain trauma for the diagnosis of post-concussional disorder [DSM-IV (6)] or PCS [ICD-10 (5)]. Differences between diagnostic systems are presented in Table 1. An important difference is that DSM-IV requires immediate symptom onset and persistence for at least 3 months whereas ICD-10 does not. In addition, DSM-IV requires objective evidence of memory or attention deficits (criterion B), but ICD-10 explicitly precludes such evidence (criterion C-3). The variability in terminology and associated criteria of the DSM-IV and ICD-10 hampers accurate identification and diagnosis of patients with PCS (13). Different classification methods may result in overestimation or underestimation of symptoms, particularly when relying on subjective endorsement of symptoms by patients. This was shown in a cross-sectional study in which 61 patients were referred to a concussion clinic following mTBI (20).

**Table 1:** Comparison of three definitions of post-concussion symptoms.

	**ICD-10**	**DSM-IV**	**DSM-5**
Headache	√	√	–
Dizziness	√	√	–
Fatigue	√	√	–
Noise intolerance	√	√	–
Irritability/lability/anxiety/depression	√	√	–
Sleep problems	√	√	–
Concentration problems	√^A^	√^B^	√^B^
Memory deficit	√^A^	√^B^	√^B^
Intolerance of alcohol	√	–	–
Preoccupation with symptoms	√	–	–
Personality change	–	√	–
Apathy	–	√	–
Perceptual-motor	–	–	√^B^
Social cognition	–	–	√^B^

*Table shows symptoms presented in the International Classification of Diseases (ICD)-10 definition of PCS (diagnosis code F07.02), the Diagnostic and Statistical Manual of Mental Disorders (DSM)-IV definition of postconcussional disorder and the DSM-V definition of neurocognitive disorder*.

A *Subjective report*.

B *Objective test*.

Post-concussional disorder was not included in the last DSM-5 edition (21). Instead, DSM-5 contains “mild neurocognitive disorder due to TBI,” a neurocognitive disorder, which strongly suggests—but does not formally require—performance-based, quantifiable evidence of acquired cognitive deficits after mTBI (Table 1). Importantly, DSM-5 denotes the status of the most frequently reported post-concussion symptoms to the level of “associated features.” Finally, DSM-5 emphasizes a broad range of differential diagnoses, especially when symptom severity “appears to be inconsistent with the severity of the TBI” (22).

### Prevalence of Post-concussion Symptoms

**Figure 2:**
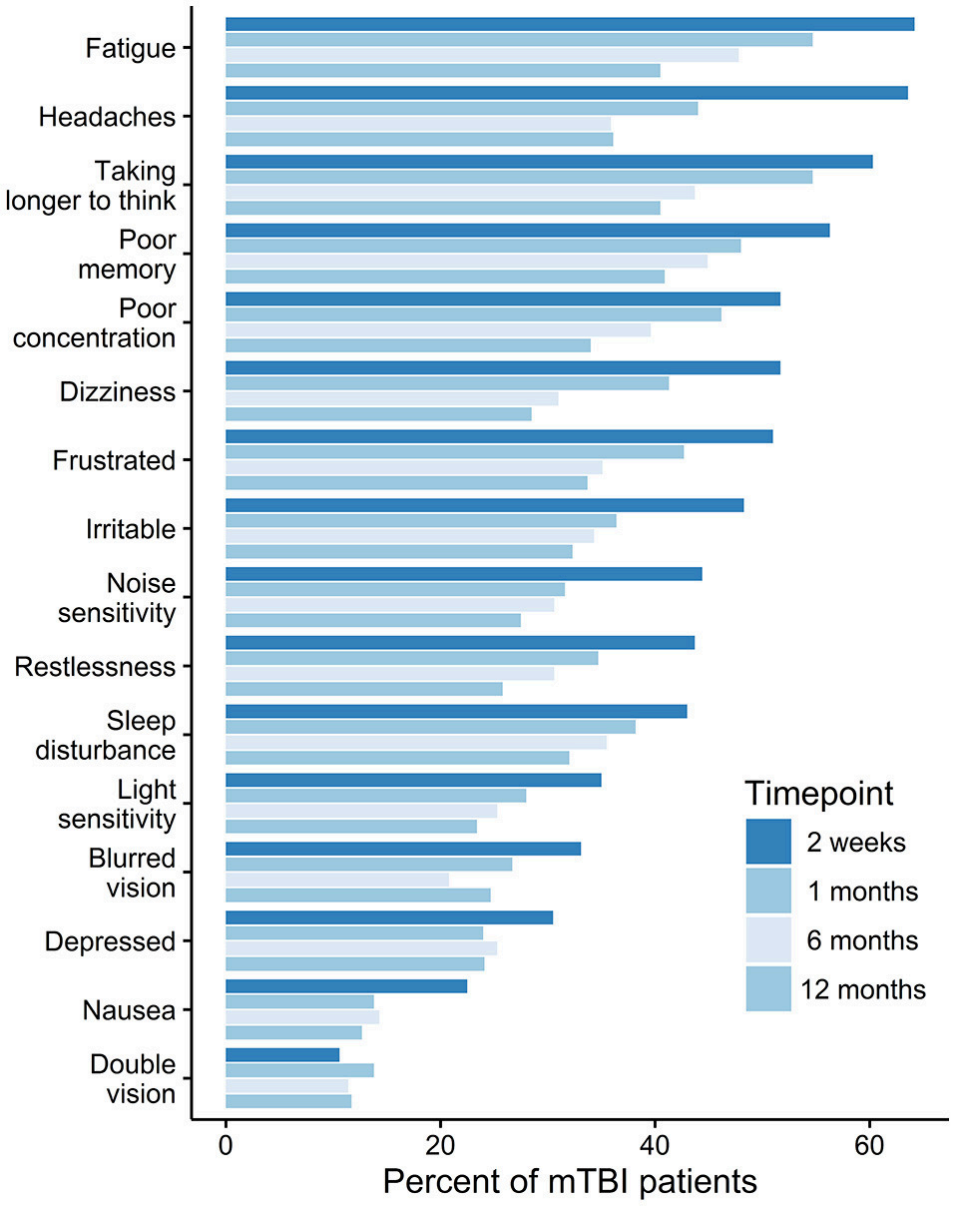
The prevalence of post-concussion symptoms over time. *Permission has been obtained to base our figure on* data presented in Theadom (27).

Prevalence of post-concussion symptoms varies and depends on pre-injury factors (10, 23), patient population (24), assessment (24), and analytic strategies, diagnostic criteria (24, 25), and classification methods (26). Overall, single symptoms (e.g., fatigue, headache, and cognitive symptoms) are very common (27) (Figure 2), whereas multiple concurrent symptoms are less frequent (24).

Neuropsychological testing consistently shows minor cognitive deficits within the first 2 weeks after injury, with some exploratory evidence suggesting deficits lasting up to 6 months (28). It has been suggested that self-reported somatic symptoms (headaches, dizziness) are more prevalent immediately after the injury (1–2 weeks) (29), whereas cognitive and emotional symptoms resolve more slowly and may still be above baseline levels long-term post-injury (30, 31). However, these cross-sectional analyses did not track the evolution of symptoms in single patient groups. Therefore, evidence supporting a differential trajectory between self-reported somatic and cognitive/emotional subacute symptoms is limited.

ICD-10 prevalence rates of PCS at 3 months post-injury vary between 6% (32), 22% (33), and 64% (25). DSM-IV diagnostic criteria appear to be stricter than ICD-10 criteria leading to lower estimates (34): a cohort study of patients after mTBI found a prevalence of PCS at 3 months of 64% based on ICD-10 criteria, but only a prevalence of 11% when using DSM-IV (25).

Few pediatric studies report on the prevalence of post-concussion symptoms according to ICD-10 or DSM-IV diagnostic criteria; 1-month prevalence for children recruited from ED based on ICD-10 reach 52% (35) and 3-month prevalence based on DSM-IV constitutes 29.3% (36). Some studies define symptomatic children as having an increase in at least one symptom and arrive at estimates between 24.5 and 52.5% at 1 month post injury (35, 37), 11–39% after 3 months, and 2.3% at 12 months (35), which makes comparison of symptom development trends between children and adults challenging. An additional complication in capturing prevalence rates in children is that younger children may not be able to describe their symptoms reliably. Therefore, such prevalence estimates should be treated with caution.

## Controversies

Post-concussion symptoms are highly controversial and a major topic of debate among clinicians, methodologists, and health outcome experts. One problem is that post-concussion symptoms do not always cluster in a consistent and predictable manner (12, 19). Therefore, it is controversial whether they truly represent a specific, cohesive, and predictable syndrome (i.e., PCS) (12, 19). In addition, although the term post-concussion symptoms might suggest otherwise, these symptoms are not specific to TBI but are also frequently reported in non-brain injured trauma patients (10), including patients with whiplash injuries (38) and in healthy adults and children (35, 39, 40).

The literature on mTBI frequently uses the term “symptom” to refer to all changes experienced after a concussion. However, when focusing solely on the patient's self-report, the use of the term “complaint” might be more appropriate.

Similarly, the etiology of post-concussion symptoms is also debatable. Although the biopsychosocial model is often applied to explain the onset and persistence of post-concussion symptoms (41), post-concussion symptoms have also been associated with malingering, exaggeration, misattribution, and recall bias, thereby prompting concern regarding the clinical reality of post-concussion symptoms.

### Acute and Persistent Post-concussion Symptoms

Acute post-injury symptoms, such as headache, dizziness, sensitivity to light or noise, double vision or tinnitus, are associated with the development of persistent symptoms (19, 42, 43). A clinical risk score in children has identified headache, sensitivity to noise, fatigue and answering questions slowly as predictive of post-concussion symptoms at 28 days post-injury (44). In addition, the experience of post-concussion symptoms early post-injury (1 week−1 month) is consistently associated with higher odds of persistent post-concussion symptoms (10, 45). A study from 2015 found that 82% out of 103 patients who were experiencing post-concussion symptoms 1 year after mTBI had already reported these 1 month post injury (46).

### Biological Factors and Persistent Post-concussion Symptoms

Several, predominantly biological factors, such as diffuse axonal injury, neuro-inflammation, and altered cerebral blood flow have been implicated in the genesis of post-concussion symptoms after mTBI (41, 47, 48). However, these factors have not yet been analyzed in high-quality prognostic studies. The role of biological factors is supported by findings that repetitive mTBI is associated with increased symptom prevalence (49, 50), longer time to symptom resolution (50, 51), and a minimal effect of neurocognitive deficits (52). Similarly, repetitive sub-concussive impacts, e.g., in contact sports, have also been associated with minor long-term neuropsychological sequelae (53), abnormalities in both neuroimaging and in neuropsychological testing (54), and with the development of severe neurodegenerative conditions such as chronic traumatic encephalopathy (CTE) (54). Although many symptoms of CTE overlap with post-concussion symptoms (e.g., irritability, impulsivity, depression, (short-term) memory loss), current evidence on the association of repetitive sub-concussive impacts with CTE is limited and should be considered preliminary (55).

A major controversy in attempting to identify the role of biological factors in the development of post-concussion symptoms is their weak relationship with injury severity and the high prevalence of PCS-like symptoms in non-brain injured patients, as well as in healthy participants (10, 35, 37–40, 45, 56).

Even though most studies report that the rate of post-concussion symptoms is higher among brain-injured patients as compared to non-brain injured trauma controls (32, 46, 57–59), the high rate of false-positives needs to be taken into account when examining biological factors. It should be acknowledged that biological factors do not exist in isolation but need to be interpreted in the context of potentially confounding factors, e.g., pre- and post-injury physical and mental health, trauma, and psychosocial factors (10, 58–60).

### Psychiatric, Psychological, (Psycho)-Social Factors and Post-concussion Symptoms

#### Psychiatric Factors

Many post-concussion symptoms (e.g., sleep difficulties, irritability and concentration problems) are similar to symptoms of the hyperarousal dimension of posttraumatic stress disorder (PTSD) (59), which may occur following exposure to severe, often life-threatening events. PTSD following mild, moderate, or severe TBI has a pooled prevalence rate of 13.8% (10.2–17.4%) (61) and appears to follow TBI more frequently than any other traumatic injuries not involving the brain (47, 62). Given the overlap between post-concussion and PTSD symptoms (59, 62, 63), careful differential diagnosis is required. Nevertheless, a prospective study including 534 brain-injured patients and 827 controls found that mTBI was a significant predictor for PTSD but not for post-concussion symptoms (59). It is not yet clear, whether these results also hold true for pediatric samples. A smaller prospective study comparing parent-reported post-concussion symptoms and PTSD symptoms in 186 children after mTBI and 99 children with non-head orthopedic injuries reported higher rates of post-concussion symptoms after mTBI but comparable rates of PTSD symptoms (63).

Almost half of patients with persistent post-concussion symptoms suffer from premorbid depression and anxiety (47, 64). Pre-injury mental health status has repeatedly been shown to predict persistent post-concussion symptoms in adult (41, 45, 47, 65) and pediatric populations (13, 35). However, the question of causality remains unclear, as psychiatric symptoms might be a reaction to experiencing persistent post-concussion symptoms, and/or mental health problems might increase the risk of reporting persisting symptoms.

#### Psychological Factors

Recall biases have been shown to influence reports of post-concussion symptoms after mTBI. Patients after mTBI expecting to experience post-concussion symptoms show higher symptom rates than patients not expecting to experience post-concussion symptoms (66). Similarly, in some patients the “good-old-days” bias may lead them to underestimate pre-injury symptoms (41, 57). If gross overrepresentation of symptoms is suspected (malingering), performance in selected neuropsychological tests can indicate whether the patient is exerting optimal effort (11, 67).

Finally, symptoms commonly occurring in everyday life, such as headache, irritability, sleep disturbance and forgetfulness may be misattributed to brain trauma (11, 33). Extensive assessments for putative somatic origins of such common symptoms may further make one believe that these symptoms are indicative of serious brain damage, leading to hypervigilance and catastrophic attributions, comparable to behaviors seen in patients with somatoform disorders or hypochondriasis (11, 33, 62, 67, 68).

#### Socio-Demographic, Social, and Personality Factors

Female sex is consistently associated with greater reporting of persistent post-concussion symptoms (45). Gender effects appear to be smaller in children (35, 37, 56). Some studies found that post-concussion symptoms are associated with lower education in adults (45) and pre-injury learning difficulties in children (37). Community integration, social support, lifestyle, and family dynamics may contribute to the development and persistence of post-concussion symptoms in adults (41, 69), and children (13, 70). However, conclusive evidence has not yet been established.

The five-factor model of Widiger and colleagues is a model of basic personality traits, consisting of five domains: neuroticism, extraversion, openness, agreeableness, and conscientiousness (71). Basic personality traits as captured in the five-factor model do not appear to be associated with persistent post-concussion symptoms (10). However, more specific traits such as high anxiety sensitivity (72), low resilience (73), coping styles (33, 74) or alexithymia (72) may be associated with persistence of symptoms. However, the cross-sectional design and small sample sizes in these studies hamper the establishment of firm conclusions in the area.

#### Predicting Persistent Post-concussion Symptoms

The identification of risk factors might be especially useful for clinical practice when combined into a prognostic model predicting patients at risk of poor outcome. However, current models are often based on small samples (9, 75) and lack internal and external validation (10, 45, 75, 76). In addition, no model is able to reliably predict outcomes at the individual patient level (45). Therefore, identification of high-risk patients might best be accomplished by careful and dense follow-up data collection. Advances in study and modeling methodology and, possibly, the incorporation of advanced imaging, and biochemical biomarkers (see Panel 1 for recommendations) may improve the ability to identify at-risk patients in the first week post-injury in the future.

PANEL 1 **Methodological recommendations for studies on post-concussion symptoms after mTBI.**

Well-designed confirmatory studies with the following characteristics have been called for to better understand post-concussion symptoms and its consequences:

- **Study design**: Prospective inception cohort studies with appropriate control group (e.g. non-brain injured patients, general population) and appropriate follow-up period to differentiate persistent deficits and symptoms due to post-concussion symptoms from the effects of pre-injury (neuro)psychiatric disorders and other non-mTBI factors. Longitudinal analyses strategies to monitor evolution of post-concussion symptoms in single patients.
- **Instruments**: use crosswalk analysis to compare incidence rates between studies using different post-concussion symptom assessment procedures. At a minimum, aim to include at least some comparable items, i.e., items whose functioning is comparable between patient samples, and evaluate other items relative to these anchor items. **Studies on predictors/prediction models** (based on Mushkudiani et al. (77) and Steyerberg (78):
  ° Sample size: N > 500
  ° Predictors should be based on theory, clinical knowledge or previous research
  ° For every predictor considered there should be at least ten cases (i.e., patients classified as having PCS)
  ° A liberal *p*-value (e.g., *p* < 0·157) (79) should be used when applying selection procedures
  ° Results should be internally validated (e.g., bootstrap validation)
  ° Both discrimination and calibration statistics should be mentioned; a score chart is warranted for implementation in clinical practice
  ° External validation: external validation in an independent dataset is a prerequisite before implementation in clinical practice. External validation and updating of an existing model should be prioritized against the development of a new model.

## Clinical Assessment of Post-concussion Symptoms

Providing optimal care depends on early and reliable identification of patients at risk of developing persistent post-concussion symptoms (12, 80) by a multidisciplinary team. Medical examination should include a history of previous TBIs, head and neck injuries, and a detailed description of the number and extent of acute concussion symptoms, preferably using standardized instruments (see Table 2). Special emphasis should also be placed on the assessment of co-morbid injuries and disorders, such as chronic headache, and other pain, cervical-disorders, visual or vestibular disorders, chronic fatigue, sleep, and somatoform disorders (35, 65, 80, 81). However, checklists alone are not sufficient to provide a *diagnosis* of persistent post-concussion symptoms as a disorder in the absence of a comprehensive multidimensional medical, neurological, and psychiatric and (neuro)psychological evaluation (64, 82).

**Table 2:** Selection of Post-concussion symptoms assessments (adults and children) based on CDE recommendations and frequent clinical use.

**Assessments**	**Examinations and instruments**	**Population**
Clinical Examination and History	Standardized medical history and history of injury event, neurological and physical examination including orientation, speech fluency, memory, concentration, dyslexia, dizziness, vertigo, sleep, cranial nerves, motor, sensory and gait assessment; balance and vestibular testing; respiratory and heart rate, blood pressure; Cervical spine range of motion and tenderness; comprehensive headache assessment; neuroimaging (if mandated by neurological deficits)	A/P
	Standardized pre- and post-injury anamnesis of depression, anxiety, stress, dissociation, behavior, and other mental health problems retro- and prospective assessment: e. g. Structured Clinical Interview-DSM, Mini International Neuropsychiatric Interview (v 5.5),	
	Diagnostic Interview Schedule for Children-IV, Neuropsychiatric Rating Schedule (NPRS), Clinician-administered PTSD Scale (CAPS)	
Self-reported post-concussion symptoms	Health and behavior inventory^*^	P
	Neurobehavioral symptom inventory^**^	A
	Post-concussion symptom inventory^**^	P
	Rivermead post-concussion symptom questionnaire^*^	A
Neuropsychological Impairments	Behavior rating inventory of executive function^**^	P
	Rey auditory verbal learning test^*^	A/P
	California verbal learning test for children^*^	P
	Delis-kaplan executive function system—verbal fluency^*^	P
	Immediate post-concussion assessment and cognitive testing^**^	A/P
	Trail making test (TMT)^*^	A
	TRAILS-PRESCHOOL^**^	P
	Cognitive battery-NIH toolbox^**^	A/P
	Wechsler abbreviated scale of intelligence^*^	P
	Wechsler adult intelligence scale^*^	A
	Wechsler intelligence scale for children-iv^*^/wechsler preschool and primary scale of intelligence -III	P
Psychological and psychiatric status	Brief-symptom-inventory-18^*^	A
	Beck-depression inventory II^**^	A/P
	Child behavior checklist^**^	P
	Patient health questionnaire-9^**^	A/P
	Screen for Child Anxiety Related Emotional Disorders (SCARED)^**^	P
	Minnesota Multiphasic Personality Inventory (MMPI)^**^	A
	Posttraumatic Stress Disorder Checklist (PCL)^**^	A
	Short Mood and Feelings Questionnaire (SMFQ)^**^	A/P
	Alcohol Use disorders identification test: self-report version (AUDIT)^**^	A
Symptom validity	Test of memory malingering (TOMM)^**^	A/P
	Medical symptom validity test^**^	A/P
Family and environment	Family Assessment Device (FAD)^**^	A/P
	Child and Adolescent Scale of Environment (CASE)^**^	P
	Family Burden of Injury Interview (FBII)^**^	P

* Common Data Elements (CDEs) recommended as basic measure;

** *CDEs recommended as supplemental measure; A, Adult TBI; P, Pediatric TBI*.

Since persistence of post-concussion symptoms has been associated with pre-, peri-, and post-injury psychological distress and risk of psychiatric disorders (PTSD, depression, anxiety, substance abuse, somatoform disorders), anamnesis should also include an assessment of pre-injury and current mental health difficulties (see Table 2) (10, 28, 61, 64). Finally, information on social and legal factors, such as availability of social support, life stressors, and involvement in legal proceedings needs to be collected (81).

A variety of symptom checklists exist to assess somatic, emotional, and cognitive post-concussion symptomatology, and require patients to indicate presence, absence, frequency, or intensity/severity of symptoms. Neuropsychological performance based outcomes include measures of attention, memory, concentration, orientation and executive function, and can corroborate subjective complaints of impaired cognition. However, cognitive deficits after mTBI are usually transient (28) and appear to be only weakly related to subjective complaints (23). Standard neuropsychological procedures should be followed to ensure that test results are not influenced by comorbid disorders [e.g., attention deficit hyperactivity disorder, and dyslexia (83, 84)], or inadequate understanding of test and questionnaire requirements, or low effort (85). Currently, only the field of sport concussion utilizes short reliable and sensitive screening instruments (7–10 min) to identify possible symptoms (86). A comprehensive overview of instruments suitable for clinical assessment is presented in Table 2. This overview is based on common data elements (CDE) recommendations (87–89) and frequent clinical use.

### Neuroimaging and Persistent Post-concussion Symptoms

**Figure 3:**
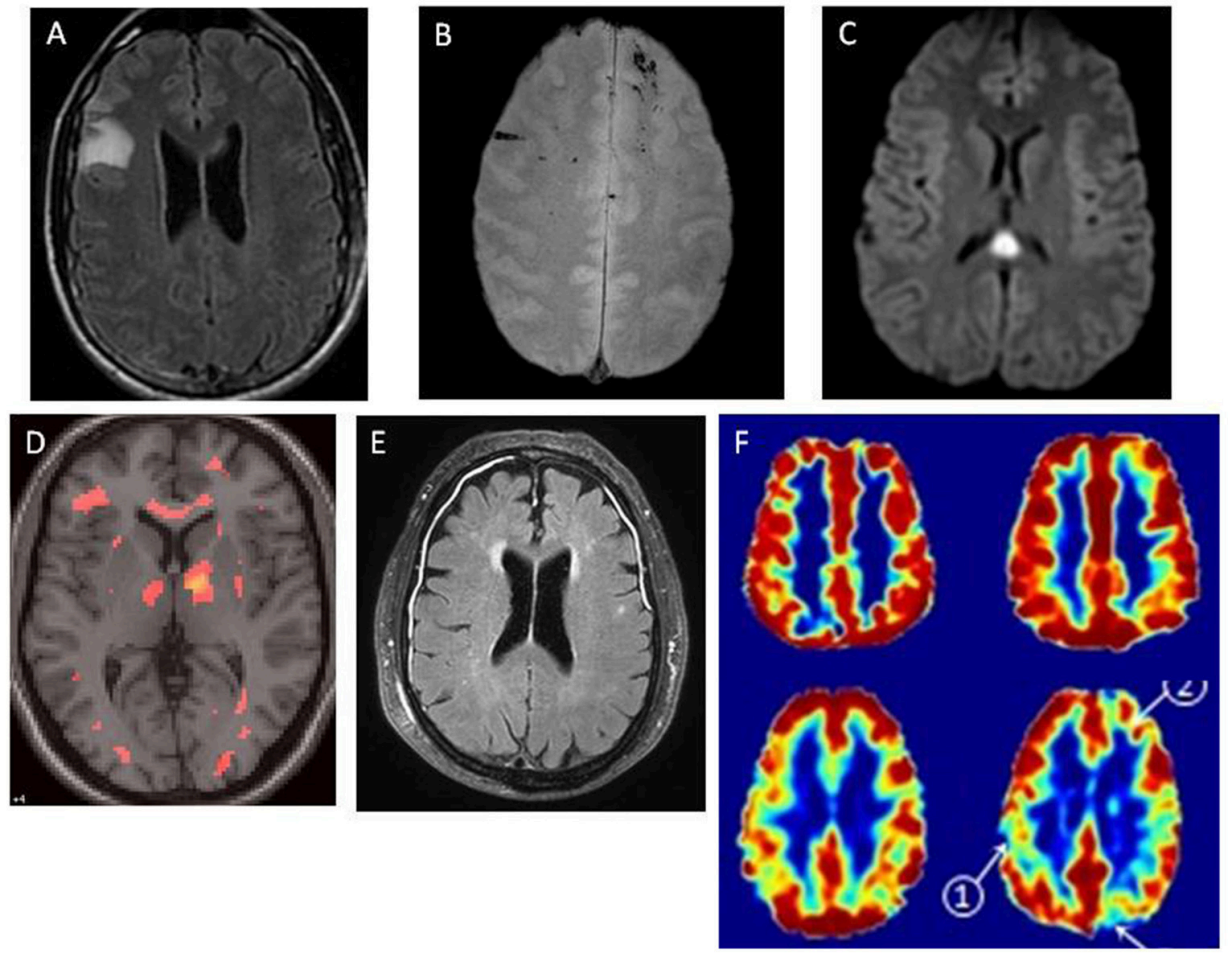
Magnetic resonance images of patients with post-concussion symptoms. MRI findings in patients with mTBI, demonstrating multiple pathologies. In each case, cranial CT was normal. MRI was obtained within 48 h on injury. **(A)** Right frontal non-hemorrhagic contusion, noted on FLAIR image. **(B)** Linear microhemorrhages in left and right frontal lobes, noted on T2^*^ image. **(C)** Diffuse axonal injury lesion in splenium of corpus callosum, with restricted diffusion noted on DWI image. **(D)** Diffuse axonal injury, with multifocal lesions noted on diffusion tensor imaging (DTI). **(E)** Traumatic meningeal enhancement of subdural effusions, noted on post-gadolinium FLAIR image. **(F)** Traumatic microvascular injury. - Top row represents a single healthy control. Bottom row represents a single TBI patient.- Left column: Cerebral Blood Flow (CBF), assessed by arterial spin labeling.- Right column: Cerebrovascular reactivity (CVR) assessed using BOLD response to hypercapnia. Credit for figures: Figures A, B, C, E: Larry Latour, PhD, NINDS/NIH; D: Carlos Marquez de la Plata, PhD, University of Texas at Dallas; F: Franck Amyot, PhD, Uniformed Services University of the Health Sciences.

No consensus has been reached on the relevance of imaging indicators of brain abnormalities for prognosis and outcome after mTBI. Figure 3 presents magnetic resonance imaging (MRI) images of patients with post-concussion symptoms. Several studies have shown that measures derived from MRI (80, 90–92) or magnetic resonance spectroscopy (MRS) can reveal structural or functional abnormalities in adults and children with an otherwise normal CT (35). Thus, for some patients, persistence of post-concussion symptoms may be explained by yet unknown brain abnormalities. However, current evidence is equivocal and the few large-scale, prognostic studies available suggest only small effects (93), if at all.

### Post-concussion Symptoms and Outcome: Health Related Quality of Life, Return to Work, and Societal Costs

Health outcome can be classified along three dimensions: health-related quality of life (HRQoL), functional, and economic outcome. Available studies suggested that post-concussion symptoms correlate with lower levels of life-satisfaction (69, 94) and HRQoL (95). HRQoL measures supplement functional and mental health outcomes with information on how health conditions influence patients' self-reports of their subjective well-being. HRQoL represents an important outcome after TBI, as it provides well-standardized information on the recovery patterns and frequency, nature, severity, and duration of the functional consequences (96). Post-concussion symptoms have been linked to lower levels of satisfaction with life (69) and HRQoL in adults (94) and children (97). However, given the association of pre-injury physical and mental health status with persistent post-concussion symptoms, the specificity of these findings is still unclear. Further research is needed to isolate the specific effects of persistent symptoms on HRQoL (14).

Furthermore, post-concussion symptoms are associated with reduced return to work (69, 98, 99). There is a need to focus on the management of persistent post-concussion symptoms to facilitate return to work (100).

The societal costs of TBI include direct medical costs and indirect expenses related to the illness and the value of lost production due to reduced working time or impaired work performance. A large part of the total lifetime costs in the field of TBI are associated with mTBI. The high incidence of mTBI, combined with a large group of patients with long-term post-concussion symptoms, results in a substantial societal and economic burden (101).

Carefully designed longitudinal research on HRQoL, functional recovery, costs and return to work is needed to differentiate persistent deficits and symptoms due to post-concussion symptoms from the effects of pre-injury neuropsychiatric disorders and other factors not associated with mTBI (14).

## Management of Patients With Post-concussion Symptoms

### Pharmacological Interventions

The evidence for pharmacological treatment of depression, anxiety, and mood lability after mTBI is limited and conflicting. A meta-analysis evaluating the effectiveness of depression treatment after mTBI found that studies using a pre-post design suggested treatment benefits from selective serotonin reuptake inhibitors (102). In contrast, the overall effects of controlled trials included in this meta-analysis did not reveal significant differences between treatment and control groups, with some evidence favoring the control condition (102). However, a recently published RCT found sertraline to be effective in preventing depression following TBI when administered early after injury (103). These findings may have considerable therapeutic implications for patients after TBI, but future studies are needed to replicate results before a change in the treatment guidelines could be recommended.

### Non-pharmacological Interventions

Evidence concerning the benefits of non-pharmacological interventions targeting post-concussion symptoms is limited. Early educational interventions in ED patients after mTBI may be promising in reducing the incidence and severity of post-concussion symptoms since a single-center RCT focusing on symptom management delivered via telephone counseling demonstrated reduced chronification of post-concussion symptoms during the first 3 months post-injury (104). This finding could not be replicated in a multi-center study; however the investigated patients showed mixed severity of TBI (105).

A recent study suggests that cognitive behavioral therapy (CBT) can improve HRQoL in patients with persistent post-concussion symptoms in the context of outpatient rehabilitation services (106). However, the effect of CBT on post-concussion symptoms was only marginal (106). Problem orientation and problem-solving skills seem to improve by neuropsychological rehabilitation addressing self-regulation of cognitive and emotional processes (107), but evidence is limited.

Evidence for beneficial effects of neuropsychological rehabilitation concerning post-concussion symptoms is limited. A systematic review found evidence that, when applied early, such approaches may be efficient in reducing self-reported post-concussion symptoms, anxiety and depression, but do not result in a clear reduction of cognitive impairment (108).

Intervention studies in children and adolescents are highly variable, of limited methodological quality, and evidence to support any particular intervention for post-concussion symptoms in pediatric samples is absent (109, 110). In adults, as in pediatric populations, well-designed prospective studies focusing on non-pharmacological multidimensional intervention that show improvement on variables such as HRQoL and return to play and work are still lacking.

### Rest and Post-concussion Symptoms

Historically, “rest” has been a foundation in the treatment of acute mTBI (70). Concerns have been raised regarding the expert-based consensus recommendation for rest after acute concussion, as studies in adults (111, 112) and children (113) indicate that prolonged rest, longer than 3 days to a week may contribute to prolonged symptomatology (114), and no reduction in post-concussion symptoms was found in a study on rest interventions (115).

### Vestibular and Vision Rehabilitation Therapy

The traumatic event resulting in mTBI may also cause concomitant cervical soft tissue damage, resulting in “whiplash-related” symptoms such as headache, dizziness, and balance dysfunction as well as cognitive, vestibular and visual dysfunction (38). A RCT comparing cervical spine physiotherapy and vestibular rehabilitation therapy (VRT) with a control condition in athletes found that among the intervention group, a significantly higher proportion of individuals were medically cleared after 8 weeks of treatment (116). However, a recent systematic review concluded that current evidence for optimal prescription and efficacy of VRT in patients after mTBI is still limited (117). In addition, large retrospective cohorts including both adults and children examining vision rehabilitation for vision disorders associated with mTBI have demonstrated clinical improvement in conditions such as convergence and accommodative insufficiency (118–120). Thus, high-level studies evaluating the effects and optimal intervention window for VRT and vision rehabilitation are required.

### Headaches

Headaches are among the most disabling symptoms after mTBI. Most post-traumatic headaches show clinical features of a recognized primary headache, such as migraine headaches or tension headaches. Post-traumatic migraines may respond to the same abortive and prophylactic treatments as sporadic migraines (121). In addition, non-pharmacological approaches such as biofeedback, physical therapy, CBT, either as primary or adjunctive treatments, have also been successfully applied to persistent post-concussion headaches (65, 122).

## Conclusions and Future Directions

Despite a sharp increase in studies investigating post-concussion symptoms, controversies and debates still exists with regard to etiology, diagnosis, classification systems, pathophysiology, natural history, prevalence, and terminology. The subjective nature of post-concussion symptoms, their low specificity, and the significant overlap with other physical, neurological, and psychiatric conditions add additional challenges to these discussions (10, 12, 19, 39, 44, 45, 56, 59, 82). The frequent overlap and idiosyncratic interplay of post-concussion symptoms with pre- and post-injury psychiatric, psychological and social factors are still under-investigated and necessitate a standardized comprehensive differential diagnosis of comorbid mental conditions, in particular depression, anxiety disorders and PTSD.

In this review, we described possible factors contributing to post-concussion symptoms from a bio-psychosocial perspective. Insights into the complex nature of post-concussion symptoms may support the risk estimate of persistent symptoms in individual patients. In addition, it may provide targets for predictive modeling which combine the different factors contributing to post-concussion symptoms. Currently, no valid model is available to predict post-concussion symptoms in adults and children (45, 75). Future predictive modeling studies could be improved by using solid methodology (see Panel 1). However, the feasibility of predictive modeling may be debated given the complex, controversial, and multifactorial nature of post-concussion symptoms. Therefore, investing in routine and economic follow-up methods (e.g., smartphone-based experience sampling approaches which have demonstrated feasibility and utility in the post-injury setting (123, 124) might be prioritized over predictive models.

The frequent reliance on simple symptom questionnaires for diagnosis ignores possible biases (10) and the fact that the major classification systems require several other criteria to be fulfilled, such as performance-based evidence of cognitive impairment (21). Most questionnaires were developed in and for patients with more severe deficits, thus their sensitivity and specificity in mTBI may be limited. More refined neuropsychological tests, especially those sensitive enough to assess cognition after mTBI, may support the diagnosis of post-concussion symptoms. Moreover, short screening batteries (computerized and paper and pencil) are needed for use in EDs and in general practice. This is aligned with international attempts at developing and implementing standards for clinical research (e.g., CDEs) (87), terminology and diagnosis criteria for post-concussion symptoms.

The heterogeneous nature of mTBI and post-concussion symptoms and the lack of reliable biological predictors and clinically useful gold-standard biomarkers (34) hamper the development of disease-modifying therapies. A first step may be the identification of specific biochemical (125) and imaging biomarkers that can complement clinical diagnosis, inform prognosis by identifying patients at risk for post-concussion symptom persistence, and predict treatment response (90, 101). Portable, lower-cost imaging modalities such as functional near infrared spectroscopy warrant further investigation to determine their clinical utility in diagnosis and management of mTBI (126).

Large-scale multidimensional, prospective longitudinal studies with several measurement points are strongly required to tackle current challenges in studying post-concussion symptoms. Such designs would allow stratified subgroup analyses to identify patients at risk for developing persistent symptoms, and might help to advance early and personalized treatment. Depending on the research question, improved designs should include control groups to provide insight into the spontaneous recovery, progression, injury severity, frequency, intensity, and fluctuation (trauma controls and healthy participants) of post-concussion symptoms.

Due to normal variation in developmental trajectories, outcomes in children after mTBI may be particularly variable. Longitudinal large sample studies (>100) that investigate predictors of post-concussion symptoms in pediatric populations with multiple endpoints, adequate controls are especially important since high neurologic and cognitive plasticity is present here.

Although evidence for effective treatments is limited, a multi-disciplinary approach corresponding to the complex etiology of post-concussion symptoms may be the most promising. Such an approach would combine in-depth comprehensive medical and neurological diagnosis with an emphasis on psychiatric differential diagnostics and psychosocial und neuropsychological outcome assessment. Future treatment directions (repetitive transcranial magnetic stimulation, vestibular and vision rehabilitation therapy, and aerobic exercise) may offer a solution for the basic pathological processes associated with post-concussion symptoms (65).

Standardization of treatment and interventions, outcome measures (87), and follow-up assessment time-points would enhance reliability and validity of research comparisons and individualized treatment. One might speculate as to whether post-concussion symptoms represent the most valid endpoint for treatment/study after mTBI. Given their low specificity, it may well be that other outcomes (e.g., functional outcome and HRQoL) prove to be more useful.

In this focused review, we only included prospective cohort studies with at least 100 participants, and reviews, with some exceptions (Appendix A). Ten included studies did not meet these criteria (20, 29, 30, 39, 40, 57, 72, 83, 84, 127). For these topics, there was no prospective study with at least 100 participants available. Therefore, prospective, multicenter research with larger patient samples is needed. In addition, it should be noted that studies fulfilling our quality criteria might still be at risk of bias. Attrition is a recurrent problem (45, 75), that may have influenced the reported prevalence rates, the relevance of etiological factors and treatment effectiveness. In addition, some studies of etiological factors were based only on univariable analyses, while multivariable assessment is highly recommended because of the multifactorial nature of post-concussion symptoms.

To summarize, standardization of the multidimensional comprehensive diagnostics, treatment interventions, and follow-up assessment time-points may enhance reliability and validity of research comparisons and refine personalized treatment and care. This review documents the need for future studies to target the identification of potential mechanisms, new imaging techniques, comprehensive multidisciplinary assessment and treatment options. Longitudinal, well controlled studies applying standardized diagnostic assessment strategies and evidence-based interventions are needed in adult and pediatric mTBI populations to optimize recovery and reduce burden of post-concussion symptoms.

## Author Contributions

SP and NvS wrote and revised the manuscript, finalized the paper based on authors, and reviewer feedback. MC conducted the search strategy, screened papers, extracted data of eligible papers, wrote, and revised the manuscript. RD-A, RR, JH, AC, CM, AG, and DV wrote and revised the manuscript. All authors critically reviewed and approved the final version of the paper.

### Conflict of Interest Statement

The authors declare that the research was conducted in the absence of any commercial or financial relationships that could be construed as a potential conflict of interest.
