# Supplementary material for: A Multidimensional Approach to Post-concussion Symptoms in Mild Traumatic Brain Injury
Source: Front Neurol. 2018 Dec 19;9:1113. doi: 10.3389/fneur.2018.01113 (PMC6306025; doi:10.3389/fneur.2018.01113)
Supplement: Supplementary file 1 [file Data_Sheet_1.docx]

**Appendix A. EMBASE search strategy**

(('postconcussion syndrome'/de OR ((postconcuss* OR post-concuss*) NEAR/3 (syndrom* OR disorder* OR symptom*)):ab,ti) AND ('prevalence'/de OR 'incidence'/de OR 'epidemiology'/de OR 'questionnaire'/de OR 'assessment of humans'/de OR 'International Classification of Diseases'/de OR 'Diagnostic and Statistical Manual of Mental Disorders'/de OR 'Rivermead behavioural inattention test'/de OR 'risk factor'/de OR 'predictor variable'/de OR 'prediction'/de OR 'prognosis'/de OR 'recurrent disease'/de OR 'recurrence risk'/de OR 'posttraumatic stress disorder'/de OR 'productivity'/de OR 'work capacity'/de OR 'return to work'/de OR 'work resumption'/de OR absenteeism/de OR 'cost of illness'/de OR 'juvenile'/exp OR (prevalen* OR incidence* OR epidemiolog* OR assessment* OR questionnaire* OR measurement* OR 'International Classification of Diseases' OR icd OR 'Diagnostic and Statistical Manual of Mental Disorders' OR dsm OR Rivermead OR risk-factor* OR predict* OR prognos* OR repetitiv* OR recurren* OR cumulat* OR 'posttraumatic stress' OR 'post traumatic stress' OR ptsd OR productivit* OR (work NEAR/3 (capacit* OR return OR resum*)) OR absenteeis* OR juvenile* OR adolescen* OR child* OR infan*):ab,ti)) OR (('concussion'/mj OR 'brain concussion'/mj OR (concuss* OR mtbi OR (mild NEAR/6 brain NEAR/3 (trauma* OR injur*)) OR ((cost OR costs) NEAR/3 (illness* OR societ*))):ti) AND ('posttraumatic pain'/mj OR 'posttraumatic headache'/mj OR 'posttraumatic stress disorder'/mj OR 'nausea and vomiting'/exp/mj OR 'dizziness'/mj OR 'headache'/mj OR 'vision'/mj OR blurred vision/mj OR 'abnormal vision'/mj OR 'fatigue'/mj OR 'insomnia'/mj OR 'sleep disorder'/mj OR sleep/mj OR 'memory'/mj OR 'memory disorder'/mj OR 'learning'/mj OR 'learning disorder'/mj OR 'attention'/mj OR 'attention disturbance'/mj OR 'concentration loss'/mj OR 'mental concentration'/mj OR 'executive function'/mj OR 'cognition'/mj OR 'depression'/mj OR 'anxiety'/mj OR 'mental instability'/mj OR 'impulsiveness'/mj OR 'emotion'/mj OR (posttraumat* OR post-traumat* OR sequel* OR nausea OR vomit* OR dizziness OR dizzy OR headache* OR vision OR visual* OR fatigue OR insomni* OR sleepless* OR sleep OR memor* OR learning OR attention OR concentration OR (executive NEAR/3 function*) OR cogniti* OR depressi* OR anxi* OR (mental* NEAR/3 instab*) OR labilit* OR impulsive* OR emotion*):ti) AND ('prevalence'/mj OR 'incidence'/mj OR 'epidemiology'/mj OR 'assessment of humans'/mj OR 'International Classification of Diseases'/mj OR 'Diagnostic and Statistical Manual of Mental Disorders'/mj OR 'Rivermead behavioural inattention test'/mj OR 'risk factor'/mj OR 'predictor variable'/mj OR 'prediction'/mj OR 'prognosis'/mj OR 'recurrent disease'/mj OR 'recurrence risk'/mj OR 'productivity'/mj OR 'work capacity'/mj OR 'return to work'/mj OR 'work resumption'/mj OR absenteeism/mj OR 'cost of illness'/mj OR 'juvenile'/exp/mj OR (prevalen* OR incidence* OR epidemiolog* OR assessment* OR questionnaire* OR measurement* OR 'International Classification of Diseases' OR icd OR 'Diagnostic and Statistical Manual of Mental Disorders' OR dsm OR Rivermead OR risk-factor* OR predict* OR prognos* OR repetitiv* OR recurren* OR cumulat* OR productivit* OR (work NEAR/3 (capacit* OR return OR resum*)) OR absenteeis* OR juvenile* OR adolescen* OR child* OR infan*):ti)) AND [2010-2016]/py NOT ([Conference Abstract]/lim OR [Conference Paper]/lim OR [Letter]/lim OR [Note]/lim OR [Editorial]/lim) AND [english]/lim NOT ([animals]/lim NOT [humans]/lim) NOT ('military phenomena'/exp OR 'blast injury'/de OR 'Alzheimer disease'/de OR 'Parkinson disease'/de OR 'dementia'/exp OR (militar* OR army OR soldier* OR iraq OR afghanistan* OR blast OR Alzheimer* OR Parkinson* OR dement*):ab,ti)
